# Quercetin Alleviates Pulmonary Fibrosis in Mice Exposed to Silica by Inhibiting Macrophage Senescence

**DOI:** 10.3389/fphar.2022.912029

**Published:** 2022-07-26

**Authors:** Fei Geng, Mengying Xu, Lan Zhao, Haoming Zhang, Jiarui Li, Fuyu Jin, Yaqian Li, Tian Li, Xinyu Yang, Shifeng Li, Xuemin Gao, Wenchen Cai, Na Mao, Ying Sun, Heliang Liu, Hong Xu, Zhongqiu Wei, Fang Yang

**Affiliations:** ^1^ Hebei Key Laboratory for Organ Fibrosis Research, School of Public Health, North China University of Science and Technology, Tangshan, China; ^2^ Hebei Key Laboratory for Chronic Diseases, School of Basic Medical Sciences, North China University of Science and Technology, Tangshan, China; ^3^ Jitang College, North China University of Science and Technology, Tangshan, China

**Keywords:** silicosis, quercetin, macrophage, senescence, SASP

## Abstract

Quercetin exerts anti-inflammatory, anti-oxidant and other protective effects. Previous studies have shown that senescent cells, such as fibroblasts and type II airway epithelial cells, are strongly implicated in the development of pulmonary fibrosis pathology. However, the role of senescent macrophages during silicosis remains unclear. We investigated the effects of quercetin on macrophage senescence and pulmonary fibrosis, and explored underlying mechanisms. Mice were randomized to six model groups. Vitro model was also established by culturing RAW264.7 macrophages with silica (SiO_2_). We examined the effects of quercetin on fibrosis, senescence-associated β-galactosidase (SA-β-Gal) activity, and senescence-specific genes (p16, p21, and p53). We showed that quercetin reduced pulmonary fibrosis and inhibited extracellular matrix (ECM) formation. Quercetin also attenuated macrophage senescence induced by SiO_2_ both *in vitro* and *in vivo*. In addition, quercetin significantly decreased the expressions of the senescence-associated secretory phenotype (SASP), including proinflammatory factors (interleukin-1α (Il-1α), Il-6, tumor necrosis factor-α (TNF-α), and transforming growth factor-β1 (TGF-β1)) and matrix metalloproteinases (MMP2, MMP9, and MMP12). In conclusion, quercetin mediated its anti-fibrotic effects by inhibiting macrophage senescence, possibly via SASP.

## Introduction

Silicosis is a chronic and progressive disease mainly caused by prolonged exposure to free silica dust, and is characterized by excessive nodular fibrosis in the lung (Hoy and Chambers, 2020; Kimberly and John, 2021). The precise underlying pathogenesis remains unclearly, therefore, comprehensive molecular investigations are required to identify treatment strategies for this disease.

Alveolar macrophages are a major cell type residing in alveoli, and function as first line defenses against inhaled infectious agents and environmental pollutants (Kim and Nair, 2019; Chen et al., 2020; Wen et al., 2021). Recent studies reported that senescent macrophages reduced proliferation, elevated senescence-associated β-galactosidase (SA-β-Gal) activity, increased senescence-associated secretory phenotype (SASP) mRNA expression, and contributed to aged disease development such as fibrosis (Sonia and Elaine, 2020; Jacques and Jesús, 2021; Su et al., 2021).

Quercetin exerts anti-oxidant, anti-inflammatory, and other protective effects (Li et al., 2016; Liang et al., 2018). Previous studies reported that quercetin reduced senescent fibroblasts and lung fibrosis in bleomycin treated mice (Liu et al., 2020; Martel et al., 2020; Li et al., 2021). Therefore, the impact of quercetin on senescence macrophages may be a key research strategy to identify mechanisms of fibrosis.

## Results

### Quercetin Treatment Effectively Alleviates Silicosis in a Mouse Model

**FIGURE 1:**
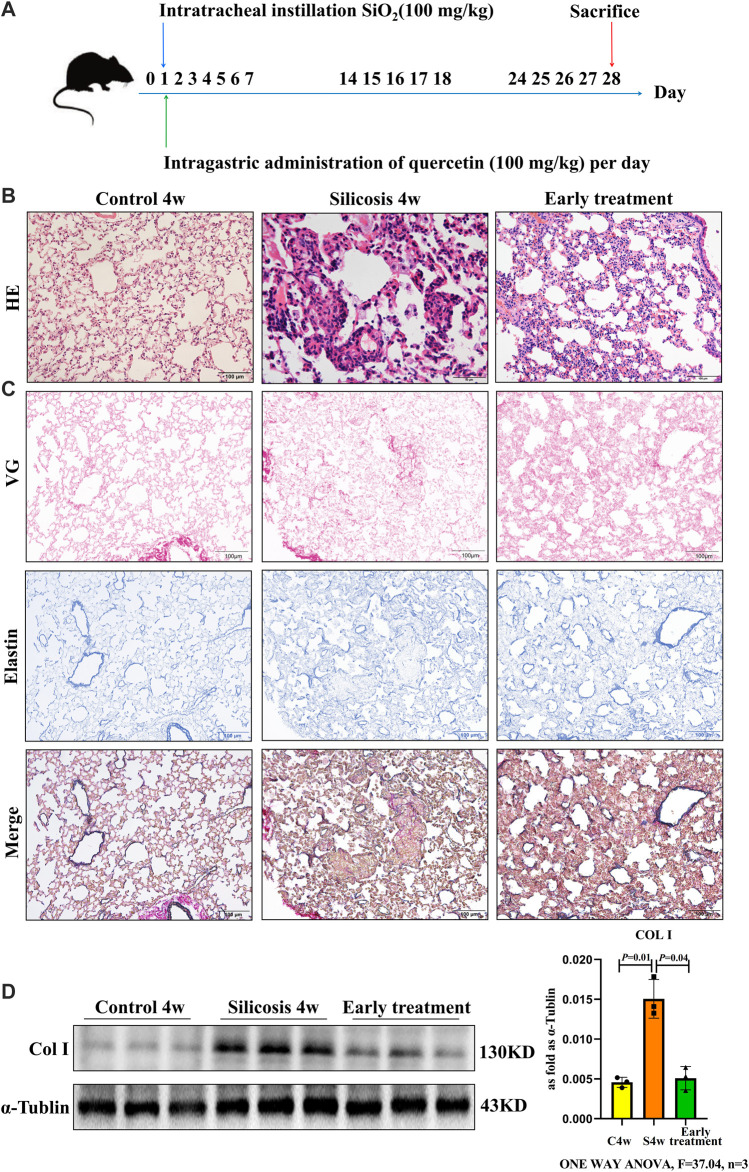
Quercetin attenuates lung injury in an early therapeutic silicosis model. **(A)** Schematic of quercetin administration in an early therapeutic silicosis model. **(B)** Representative micrographs showing lung injury by HE staining. **(C)** VG and elastin staining of lung tissue sections. **(D)** Col I expression by western blot. Data are presented as the mean ± SD. *n* = 3 per group.

To establish an early therapeutic model of quercetin administration, mice were intragastric administration of quercetin for 4 weeks at the time of silica (SiO_2_) via one-off non-invasive instillation (Figure 1A). From hematoxylin and eosin (HE) staining, more infiltrating inflammatory cells and silicotic nodules were observed in silicosis group at 4 weeks. These alterations were ameliorated after treatment with quercetin (Figure 1B). After silica exposure, the expression of the fibrotic indicator, collagen was examined in lung tissues. Van Gieson (VG) and elastin staining showed excessive collagen deposition in pulmonary mesenchyme in silicosis group. Nonetheless, collagen deposition areas were diminished after quercetin administration (Figure 1C). Western blot showed that collagen I (Col Ⅰ) levels were significantly higher in silicosis group compared with control group. In quercetin treatment group, a marked decrease of Col Ⅰ levels was observed (Figure 1D). Thus, early therapeutic administration of quercetin ameliorated silicosis *in vivo*.

**FIGURE 2:**
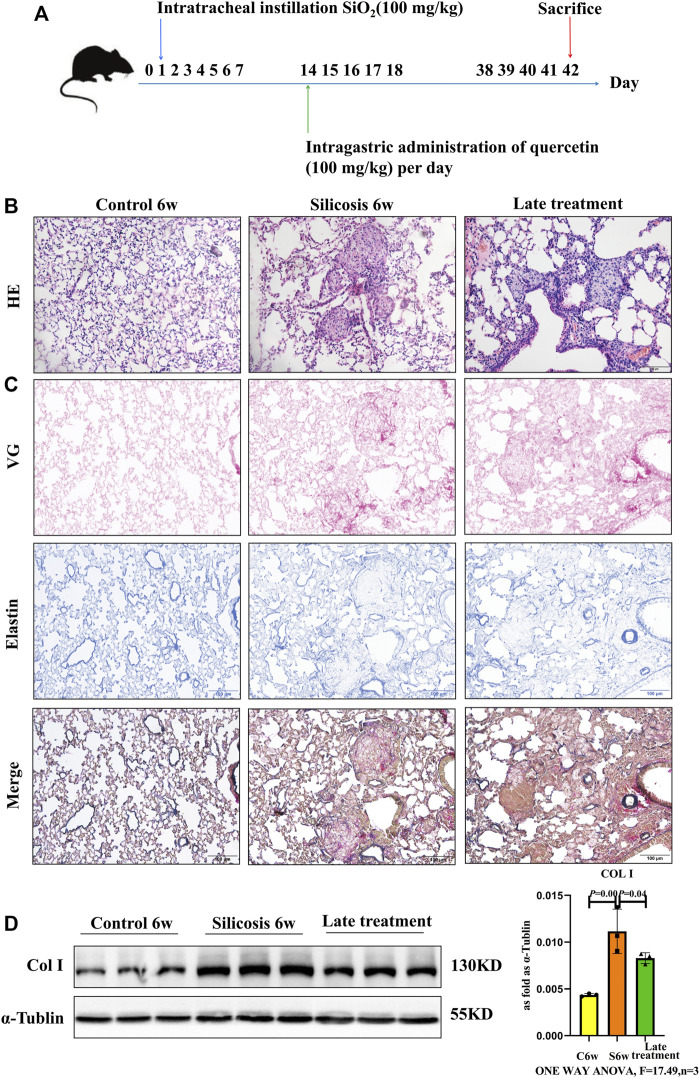
Quercetin attenuates lung injury in a late therapeutic silicosis model. **(A)** Schematic of quercetin administration in a late therapeutic silicosis model. **(B)** Representative micrographs showing lung injury by HE staining. **(C)** VG and elastin staining of lung tissue sections. **(D)** Col I expression by western blot. Data are presented as the mean ± SD. *n* = 3 per group.

We next investigated the late therapeutic model of quercetin administration after the formation of silicotic nodules in silicosis group, and mice were also intragastric administration of quercetin for 4 weeks beginning at 2 weeks after SiO_2_ exposure (Figure 2A). HE staining showed the anti-fibrotic effects of quercetin treatment for the onset of silicotic nodules (Figure 2B). In addition, reduced Col Ⅰ levels by western blot were consistent with the results using VG and elastin staining (Figures 2C,D). Collectively, these data suggest that quercrtin has improved effects on pulmonary injury when administered after onset insilicosis mice alsoly, and subsequent mechanism experiments were also conducted in this late therapeutic silicosis model.

### Quercetin Ameliorates Macrophage Senescence in Mice With Silicosis

**FIGURE 3:**
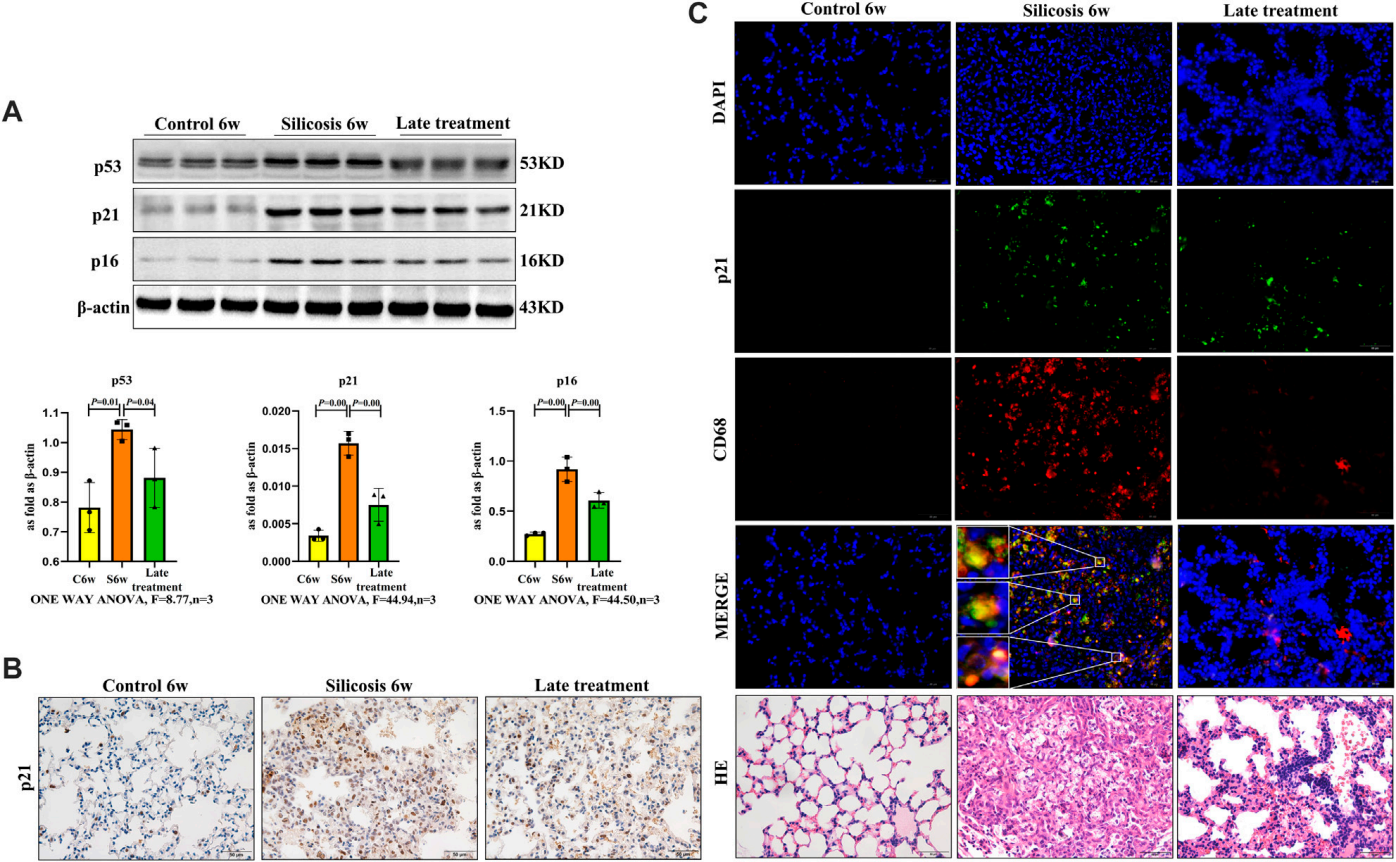
Quercetin attenuates macrophage senescence in silicosis model. **(A)** p16, p21 and p53 expression by western blot. **(B)** Immunohistochemistry staining of p21 in the lung. **(C)** Immunofluorescence staining for p21 and CD68 in the lung. Data are presented as the mean ± SD. *n* = 3 per group.

Western blot showed that quercetin administration downregulated the higher level of senescence markers, p16, p21 and p53 in silicosis group (Figure 3A).

While previous studies showed that mice exposed to SiO_2_ induced a significant increase in senescent cells and contributed to silicosis, the role of senescent macrophages requires clarification. To further assess if quercetin was associated with reduced macrophage senescence and mediated in silicosis, the senescence marker-p21 and macrophage marker-CD68 were used to examine macrophages senescence in silicosis. We observed increased numbers of p21^+^\CD68^+^ cells after SiO_2_ administration, thereby supporting the role of macrophages in senescence-associated silicosis. The percentage of p21^+^macrophages was significantly decreased in the quercetin group compared with the silicosis group (Figures 3B,C). Thus, quercetin treatment inhibited SiO_2_-induced macrophage senescence.

### SiO_2_ Induces Macrophage Senescence in a Time- and Dose-Dependent Manner

**FIGURE 4:**
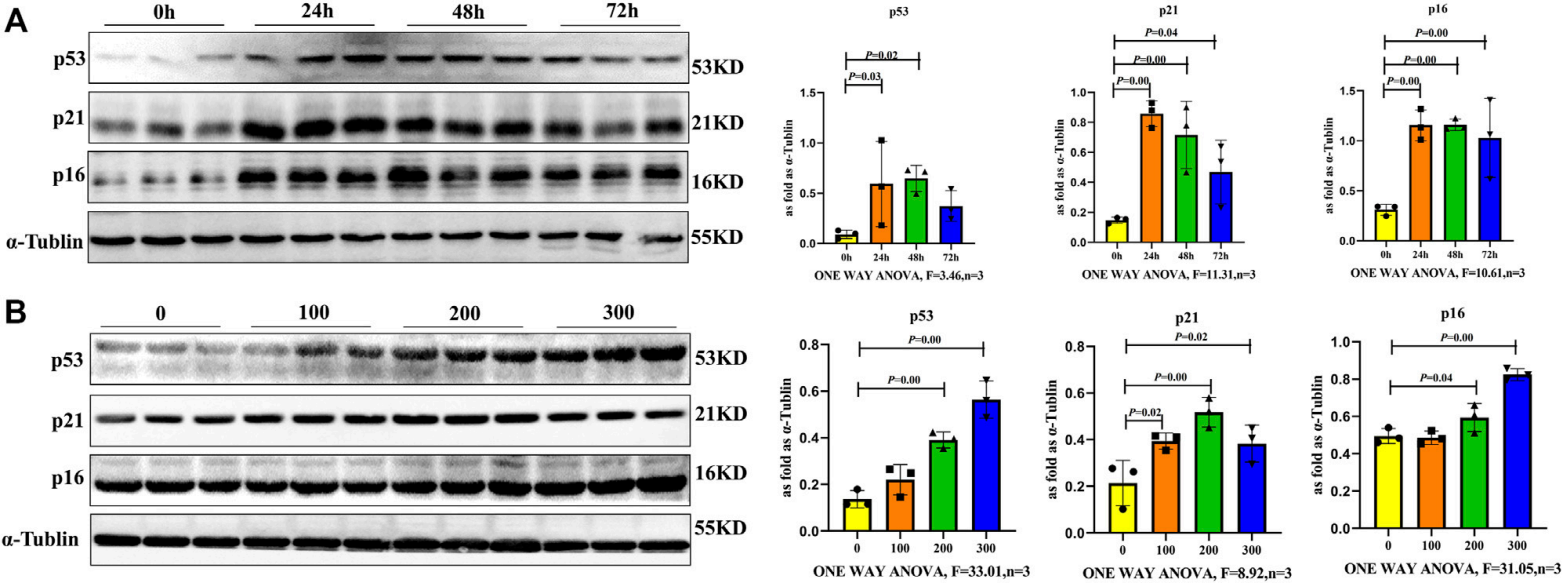
Macrophages exhibited a senescent phenotype after SiO_2_ exposure. **(A)** p16, p21 and p53 expression levels in RAW264.7 cells following 200 μg/ml SiO_2_ exposure for 24, 48 and 72 h by western blot. **(B)** p16, p21 and p53 expression levels in RAW264.7 cells following SiO_2_ exposure (100, 200, or 300 μg/ml) for 24 h by western blot. Data are presented as the mean ± SD. *n* = 3 per group.

To further verify if SiO_2_ induced macrophage senescence, RAW264.7 cells were examined at different times after 100, 200 or 300 μg/ml SiO_2_ exposure. Senescence-associated proteins of p16, p21 and p53 were gradually upregulated in a time- and dose-dependent manner. The levels of p16, p21 and p53 induced by the 200 μg/ml SiO_2_ displayed higher expression in 24 and 48 h groups compared with control (Figure 4A). In addition, the levels of p16, p21 and p53 for 24 h displayed higher expressions in 200 μg/ml and 300 μg/ml groups compared with control (Figure 4B). Thus, macrophages could be induced senescent by 200 μg/ml SiO_2_ after 24 h, suggesting senescent macrophages may be involved in silicosis development.rcetin Inhibits SiO_2_-Induced Macrophage Senescence

**FIGURE 5:**
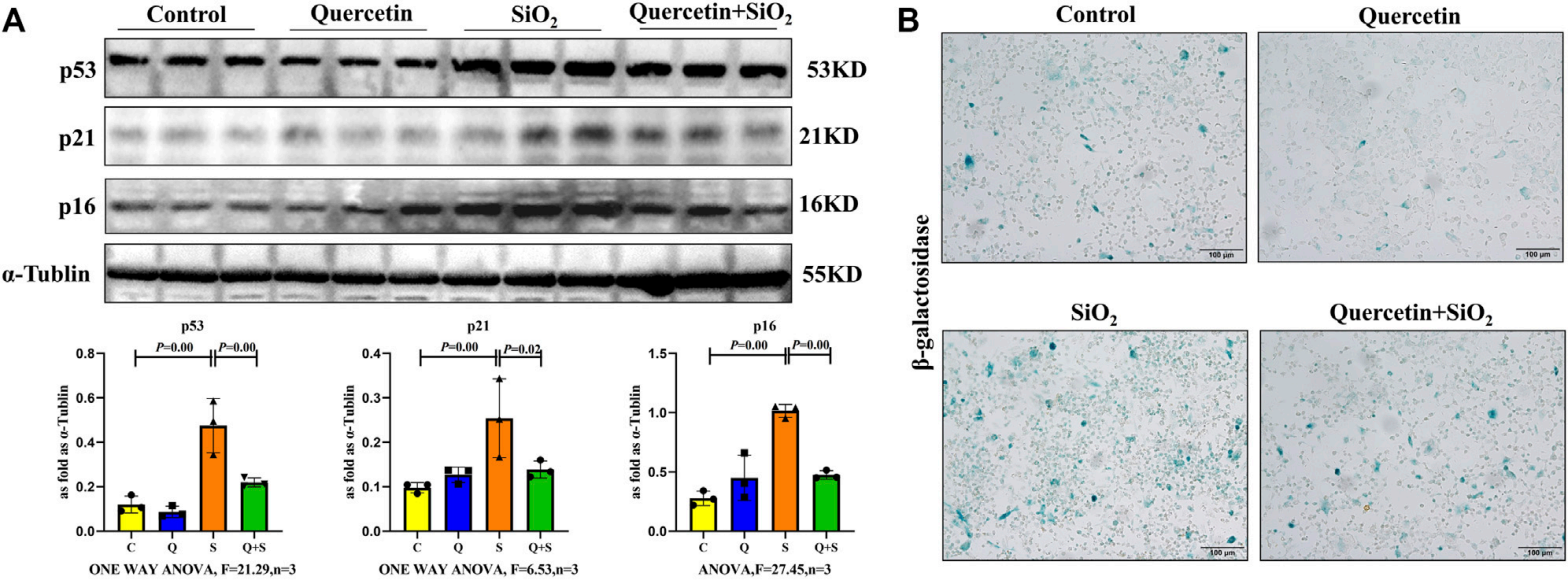
Quercetin inhibits SiO_2_-induced macrophage senescence. **(A)** p16, p21 and p53 expressions by western blot. **(B)** SA-β-gal staining in RAW264.7 cells. Data are presented as the mean ± SD. *n* = 3 per group.

To investigate the effect of quercetin on SiO_2_ induced macrophage senescence, RAW264.7 cells were treated with SiO_2_ (200 μg/ml) and/or quercetin (25 μmol/L) for 24 h. As shown in Figure 5A, the expressions of p16, p21, and p53 were significantly increased by SiO_2_ stimulated for 24 h, however, p16, p21, and p53 expressions were decreased after quercetin treatment. Cytoplasmic SA-β-gal activity, which is a marker of cell senescence, was examined by SA-β-gal staining. These assays showed that positive staining cell numbers in Quercetin + SiO_2_ group were significantly lower than SiO_2_ group (Figure 5B), confirming our western blot findings. Thus, quercetin administration significantly delayed macrophage senescence induced by SiO_2_.

### Network Pharmacology Analysis of a Quercetin-Senescence-Silicosis Intersection Target Network

**FIGURE 6:**
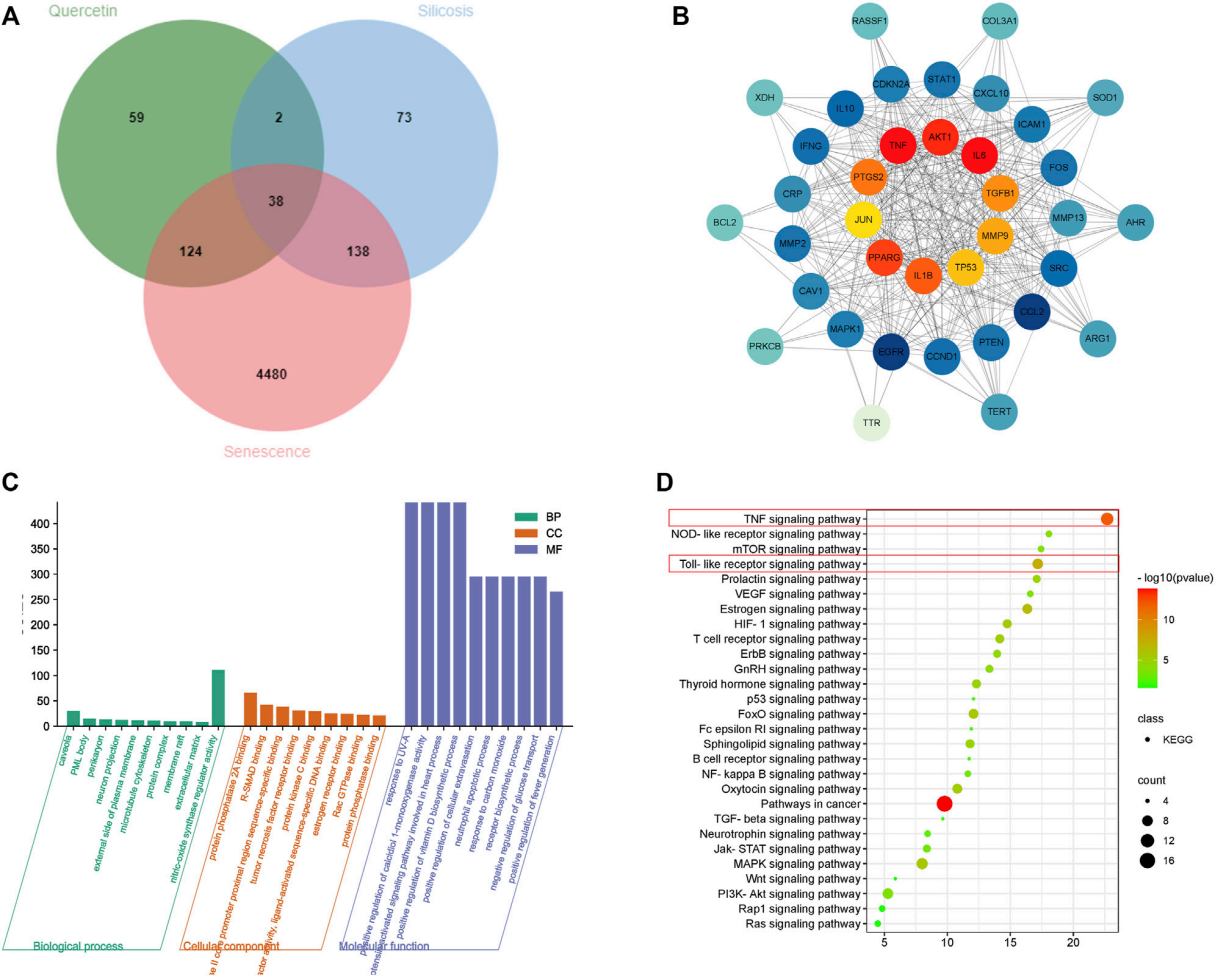
Network pharmacology analysis of a quercetin-senescence-silicosis intersection target network. **(A)** Venn diagram showing a quercetin-senescence-silicosis intersection target network. **(B)** PPI network of quercetin-senescence-silicosis intersection targets. **(C)** GO enrichment analyses. **(D)** KEGG enrichment analyses showing the top 10 pathways.

To clarify quercetin mechanisms related to senescence, we performed a network pharmacology analysis. In total, 223 potential quercetin targets were identified using Traditional Chinese Medicine Systems Pharmacology Database and Analysis Platform (TCMSP) and Swiss Target Prediction Database. We also identified 251 silicosis disease targets in GeneCards and Diagene. The GeneCards Database revealed 4,780 potential targets for senescence. Using a Venn diagram of quercetin, silicosis and senescence target intersections, 38 overlapping targets were identified (Figure 6A).

These 38 targets were then imported into the STRING Database to establish a protein-protein interaction (PPI) network. Next, cytoscape software was used to generate a quercetin-senescence-silicosis target network diagram, showing the top 10 molecular targets: interleukin-6 (IL-6), protein kinase B (AKT1), tumor necrosis factor (TNF), prostaglandin-endoperoxide synthase 2 (PTGS2), peroxisome proliferator activated receptor gama (PPARG), IL-1β, transforming growth factor-β1 (TGF-β1), matrix metalloproteinases-9 (MMP9), TP53, and c-Jun N-terminal kinase (JUN) (Figure 6B).

After gene ontology (GO) and Kyoto Encyclopedia of Genes and Genomes (KEGG) analyses, 343 biological processes, 34 molecular functions, 26 cell components and 28 signal pathways were identified (Figures 6C,D). According to adjusted *p* values, the top ten pathways were selected, including pathways in cancer, TNF signaling pathway, Toll-like receptor signaling pathway, Estrogen signaling pathway, Mitogen-activated protein kinase (MAPK) signaling pathway, Forkhead box protein O (FoxO) signaling pathway, Hypoxia inducible factor 1(HIF-1) signaling pathway, oxytocin signaling pathway, T cell receptor signaling pathway, and thyroid hormone signaling pathway.

### The Senescent Macrophage Secretome Is Profibrotic and Proinflammatory

**FIGURE 7:**
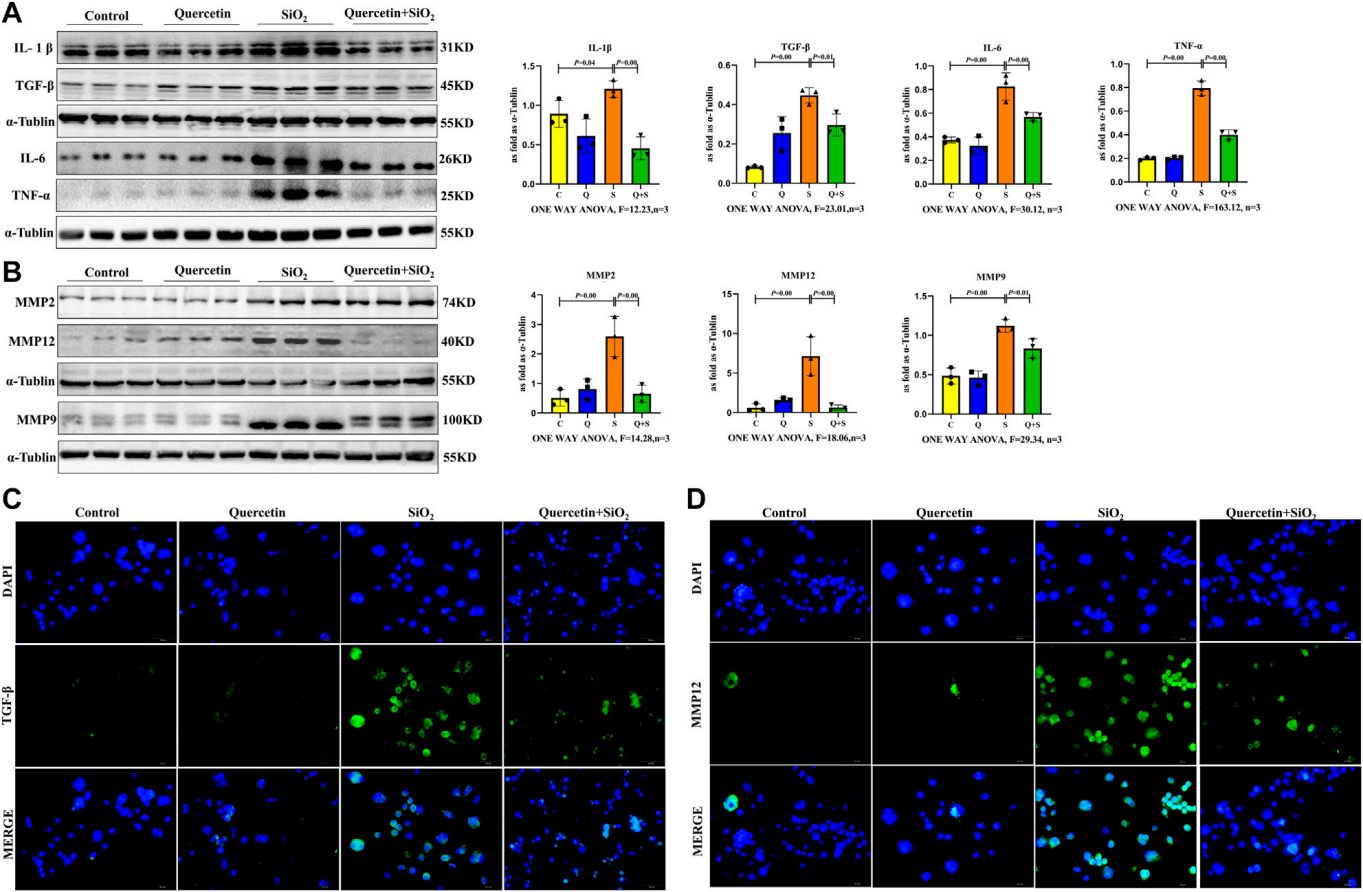
Elevated fibrotic and SASP protein expression in senescent macrophages. **(A)** IL-1β, IL-6, TNF-α and TGF-β1 expressions by western blot. **(B)** MMP2, MMP9 and MMP12 expressions by western blot. **(C)** Immunofluorescence staining for TGF-β1. **(D)** Immunofluorescence staining for MMP12. Data are presented as the mean ± SD. *n* = 3 per group.

To explore the role of senescent macrophages during silicosis, dynamic changes in SASP components were determined after macrophages were exposed to SiO_2_, including the analysis of growth and matrix remodeling factors. Firstly, the SASP proinflammatory factors, including IL-1β, IL-6, TNF-α, and TGF-β1 were significantly upregulated in senescent macrophages at 24 h after 200 μg/ml SiO_2_ exposure (Figures 7A,C). Secondly, MMPs including MMP2, MMP9 and MMP12 of the SASP matrix in macrophages were increased after macrophages exposure to SiO_2_ (Figures 7B,D). Taken together, our results supported the notion that SiO_2_ promoted SASP secretion in senescent macrophages.

Importantly, SASP proinflammatory factor secretion and MMPs in the SASP matrix were decreased after quercetin treatment. (Figure 7).

### Quercetin Alleviates Silicosis by Reducing SASP Levels of Senescent Macrophages in a Silicosis Mouse Model

**FIGURE 8:**
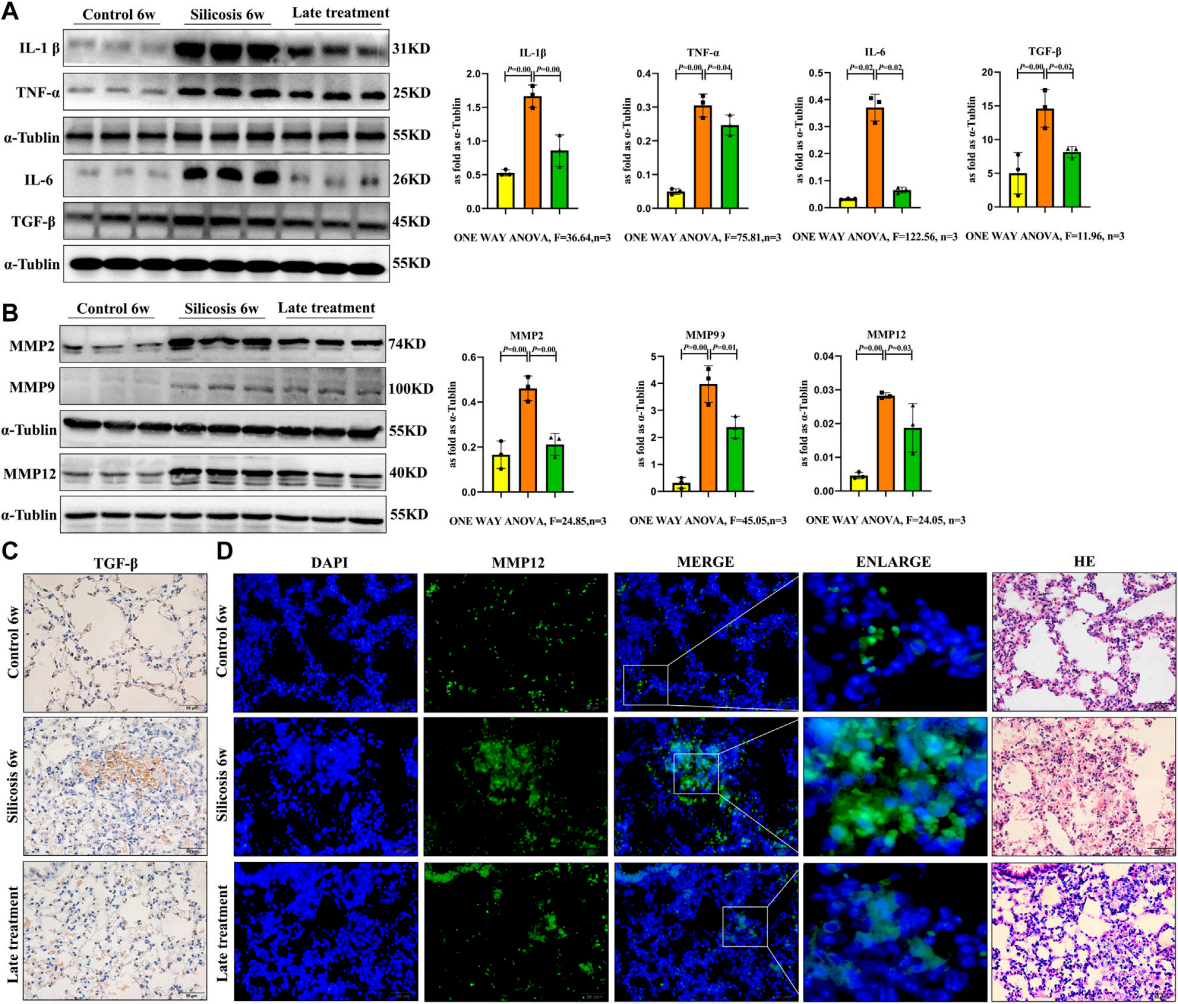
SiO_2_ significantly elevated fibrotic and SASP protein expression levels in the lung tissue of silicosis model. **(A)** IL-1β, IL-6, TNF-α and TGF-β1 expression levels were detected by western blot. **(B)** MMP2, MMP9 and MMP12 expression levels were detected by western blot. **(C)** Immunohistochemistry staining for TGF-β1 in the lung. **(D)** Immunofluorescence staining for MMP12 in the lung. Data are presented as the mean ± SD. *n* = 3 per group.

Fibrotic and SASP protein expression levels in the lungs of the silicosis model were analyzed. Western blot showed that the expression levels of IL-1β, IL-6, TNF-α and TGF-β1 in lung tissue from the silicosis group were significantly elevated (Figures 8A,C). Additionally, increased MMP2, MMP9 and MMP12 expression levels were also detected after SiO_2_ exposure (Figures 8B,D).

Quercetin treatment significantly decreased fibrotic and SASP protein expression levels *in vivo*. Thus, quercetin administration inhibited silicosis activity by attenuating SASP levels in senescent macrophages in a silicosis model.

## Discussion

Silicosis has a chronic, progressive and ultimately fatal fibrotic course, thus effective preventative and therapeutic measures are required. The disease is characterized by fibroblast proliferation, ECM accumulation, and ultimately leading to respiratory failure (Wei et al., 2019; Li et al., 2020). Although the disease pathogenesis has been widely investigated, detailed molecular and cellular pathogenic mechanisms are unclear with current therapeutic strategies largely ineffective.

Quercetin is a natural plant flavonoid in many fruits, vegetables and grains, which displays numerous beneficial biological activities including anti-inflammatory, anti-oxidant, immune regulation and other effects (Fontana and Partridge, 2015). Quercetin suppressed bleomycin induced epithelial-mesenchymal transition by suppressing reactive oxygen species via Smad and β-catenin signaling pathways (Takano et al., 2020). Quercetin also exerted anti-fibrogenic and anti-inflammatory effects, possibly by modulating the redox balance via nuclear erythroid factor 2-related factor 2 (Nrf2) induction (Boots et al., 2020). Quercetin (100 mg/kg) markedly reduced the extent of tissue damage in bile duct ligation (BDL) - and carbon tetrachloride (CCl4) -liver fibrosis model mice, confirming its protective effect (Wu et al., 2017). In our study, we showed that both early and late therapeutic administration of quercetin (100 mg/kg) protected against SiO_2_-induced silicosis in mice.

Cellular senescence involves DNA damage responses which activate the tumor suppressors including p16, p21 and p53, and the subsequent induction of SA-β-gal, which has key roles in embryogenesis, wound healing and protection against cancer (Birch et al., 2017). Previous studies reported that senescent cells including senescent fibroblasts and type II airway epithelial cells are contributed to the development of pulmonary fibrosis and (Schafer et al., 2017). Pulmonary macrophages are predominant effector cells with vital roles in lung fibrosis pathogenic mechanisms. However, the precise role of these macrophages remains largely unexplored. Here, we showed that similar trait combinations were acquired by macrophages following SiO_2_ exposure both *in vivo* and *in vitro*. In the silicosis model, SiO_2_ increased the expressions of the senescence-specific proteins, p16, p21 and p53 in lung tissue, and numbers of p21-expressing macrophages by immunohistochemistry staining. *In vitro*, RAW264.7 cell elevated SA-β-Gal activity after SiO_2_ exposure, suggesting pulmonary macrophages exhibited senescence characteristics during silicosis.

Quercetin inhibited phosphatidylinositol-3-hydroxykinase (PI3K) and Akt to reduce senescent cells and delay aging as indicated by improved cardiac function and exercise capacity (Zhu et al., 2015). Quercetin (20 µmoL/L) also alleviated kidney fibrosis by reducing renal tubular epithelial cell senescence via mitophagy, possibly by activating the Sirtuin-1(SIRT1)/PTEN-induced kinase 1 (PINK1)/Parkin axis (Liu et al., 2020). 25 µmoL/L of quercetin inhibited the formation of foam cells induced by oxidized low density lipoprotein (ox-LDL) and delayed RAW264.7 cell senescence (Cao et al., 2019). Our findings showed that quercetin (25 µmoL/L) effectively delayed macrophage senescence after SiO_2_ exposure, thereby confirming the anti-silicosis and anti-aging effects of quercetin.

Generally, senescent cells contribute to tissue dysfunction via SASP, which comprises pro-inflammatory cytokines (e.g., IL‐1β and IL‐6) and MMPs (Daniel and Manuel, 2014). Senescent cells and SASP may fuel increasingly fibrotic responses, driving lung deterioration and severe functional decline during silicosis.

To screen for key quercetin targets against senescence and silicosis, our network pharmacology approach identified 38 overlapping targets, with 10 hub genes identified: IL-6, AKT1, TNF, PTGS2, PPARG, IL-1β, TGF-β1, MMP9, TP53 and JUN. Based on KEGG pathway analyses of target proteins, both TNF and Toll-like receptor signaling pathways were related to senescence responses and silicosis. Thus, TNF, IL-6, IL-1β, TGF-β1 and MMP9 modulation may be key pharmacological mechanisms underpinning quercetin action against senescence. Our findings support the hypothesis that SiO_2_ induced macrophage senescence and promoted the SASP secretion of profibrotic factors, chemokines and MMPs leading to irreversible lung fibrosis.

Also, SiO_2_ induced macrophage senescence and silicosis in lung tissue, both of which were accompanied by increased fibrosis-associated factors (IL-1β, IL-6, TNF, and TGF-β1) and MMPs (MMP2, MMP9 and MMP12). Importantly, quercetin appeared to restrain SASP secretion.

## Materials and Methods

### Animal Studies

Animal studies were approved by the Institutional Animal Care and Use Committee of the North China University of Science and Technology. Animals received *ad libitum* access to food and water according to the guidelines of the National Institutes of Health (NIH, MD, United States).

Female C57BL/6 mice (6–8 weeks old) were purchased from Beijing HFK Bioscience Co. Ltd. (Beijing, China). The silicotic model was established by given silica (SiO_2_) (100 mg/kg) *via* one-off non-invasive intratracheal instillation. The quercetin therapeutic group was intragastric administration of quercetin (100 mg/kg) once a day.

To establish both early and late therapeutic model of quercetin administration, sixty mice were randomly divided into six groups: 1) Control 4w group (placebo treatment for 4 weeks); 2) Silicosis 4w group (given SiO_2_ for 4 weeks); 3) Quercetin 4w groups (given SiO_2_ and simultaneously administration of quercetin for 4 weeks); 4) Control 6w group (placebo treatment for 6 weeks); 5) Silicosis 6w group (given SiO_2_ for 6 weeks); 3) Quercetin 6w groups (given SiO_2_ and administration of quercetin for 4 weeks beginning at 2 weeks after silica exposure).

Mice were humanely anaesthetized, and sera and lung samples collected and frozen at −80°C until required.

### Cell Culture

RAW264.7 mouse macrophages were purchased from the Chinese Academy of Sciences Cell Library (Shanghai, China). Cells were cultured in F12 K media (BOSTER; California, United States) containing 10% fetal bovine serum (Bovogen Biologicals; Melbourne, Australia) at 37°C in 5% CO_2_. After 8 h starvation in serum-free F12K, cells were randomly divided into four groups: Control; Quercetin (25 μmol/L); SiO_2_ (200 μg/ml); Quercetin + SiO_2_ (added of quercetin (25 μmol/L) for 2 h before SiO_2_ (200 μg/ml) stimulation).

### Histology

Briefly, lung tissue slides were stained in HE to assess silicosis. VG and elastin stains were used to assess collagen fiber deposition.

### Western Blot

Total protein was extracted from lung tissue or cells using RIPA buffer (Cat. No. JFR0150; JFBiotech, Shanghai, China) containing a protease inhibitor (Cat. No. RP-WA0102; Report Biotech, China). Protein concentrations were measured using the Bradford assay (Cat. No. RP-WA0201; Report Biotech). Then, 15 μg protein was separated using sodium dodecyl sulfate-polyacrylamide gel electrophoresis on 10% gels. Proteins were transferred to polyvinylidene difluoride membranes (Sigma-Aldrich; Shanghai, China) and membranes blocked in 5% non-fat milk at room temperature for 1 h. Then, membranes were incubated overnight at 4°C with primary antibodies (anti-collagen type I [Cat. No. Ab34710; Abcam, Cambridge, United Kingdom], anti-p16 [Cat. No. A0262; Abclonal, Wuhan, China], anti-p21 [Cat. No. 5556431; BD Pharmingen, Guangzhou, China], anti-p53 [Cat. No. SC-6243; Santa Cruz Biotechnology, CA, United States], anti-interleukin-1α (anti-IL-1β) [Cat. No. DF6251; Affinity, Cincinnati, OH, United States], anti-IL-6 [Cat. No. A0286; Abclonal], anti-tumor necrosis factor-α (anti-TNF-α) [Cat. No. A11534; Abclonal], anti-transforming growth factor-β1 (anti-TGF-β1) [Cat. No. ARG56429; Arigo, Taiwan, China], anti-matrix metalloproteinase 2 (anti-MMP2) [Cat. No. AF0577; Affinity], anti-MMP9 [Cat. No. AF5228; Affinity], and anti-MMP12 [Cat. No. ET1602-42; HUABIO, Hangzhou, China]).

Membranes were washed three times in TBS-T and incubated with diluted (1:5,000) peroxidase-labelled affinity-purified antibodies targeting rabbit/mouse IgG (H + L) (Cat. No. 5220-0336(074-1506) and Cat. No. 5220-0341(074-1806); Seracare, United States) at 37°C for 1 h. After washing three times in TBS-T, membranes were visualized by adding enhanced chemiluminescent Prime Western Blot Detection Reagent (Cat. No. ZD310A-1; ZOMANBIO, China). Grey values were analyzed using ImageJ software (NIH). α-Tubulin and β-actin was used as a loading control.

### Immunocytochemistry and Immunohistochemistry

These techniques were performed on lung tissue sections and RAW264.7 cells. Non-specific binding sites were blocked for 30 min in blocking solution (phosphate-buffered saline (PBS) plus 1% bovine serum albumin). Samples were incubated overnight at 4°C with primary antibodies against p21 and TGF-β1, and then incubated with secondary antibodies (Cat. Mo. PV-6000; ZSGB-BIO, Beijing, China) at 37°C for 30 min. Immunoreactivity was visualized with diaminobenzidine (Cat. No. ZLI-9018; ZSGB-BIO). Brown staining was considered a positive result.

### SA-β-Gal Staining

SA-β-gal activity staining was performed as per manufacturer’s instructions (Cat. No. C0602; Beyotime, Shanghai, China). Briefly, cells were washed in PBS (pH 7.2), fixed in fixative solution (provided by the kit), and stained in a 5-bromo-4-Chloro-3-Indolyl β-d-Galactopyranoside (X-gal) solution for 48 h at 37°C. Imaging was performed using bright-field microscopy where senescent cells stained blue.

### Network Pharmacologic Analysis

Identify protein targets of quercetin were used TCMSP (http://tcmspw.com/)and Swiss Target Prediction (http://www.swisstargetsprediction.ch/). Targets for silicosis and senescence were collected from Gene Cards database (https://www.genecards.org/) and Disgene (http://www.disgenet.org/). The PPI network of these targets was obtained from the String (https://string-db.org). Finally, we used Cytoscape software to open the results, created quercetin-silicosis-senescence targets network.

### Statistical Analysis

Statistical analyses were conducted in SPSS statistical software and performed with one‐way analysis of variance, followed by *post-hoc* analysis with Bonferroni’s tests for multiple comparisons. Data were presented as the mean ± standard deviation. *p* values <0.05 were statistically significant.

## Conclusion

In summary, we suggest that senescent macrophages and SASP mechanisms may be treatment targets for silicosis, with quercetin functioning to eliminate senescent macrophages and block SASP activity, thereby acting as a novel therapeutic approach for silicosis.

## Data Availability

The original contributions presented in the study are included in the article/Supplementary Material, further inquiries can be directed to the corresponding authors.
